# Surface Plasmon Resonance Optical Sensor: A Review on Light Source Technology

**DOI:** 10.3390/bios8030080

**Published:** 2018-08-26

**Authors:** Briliant Adhi Prabowo, Agnes Purwidyantri, Kou-Chen Liu

**Affiliations:** 1 Research Center for Electronics and Telecommunications, Indonesian Institute of Sciences, Bandung 40135, Indonesia; 2 Department of Electronics Engineering, Chang Gung University, Taoyuan 33302, Taiwan; 3 Research Unit for Clean Technology, Indonesian Institute of Sciences, Bandung 40135, Indonesia; agnes.purwidyantri@lipi.go.id; 4 Division of Pediatric Infectious Disease, Department of Pediatrics, Chang Gung Memorial Hospital, Taoyuan 33305, Taiwan; 5 Department of Materials Engineering, Ming Chi University of Technology, New Taipei City 24301, Taiwan

**Keywords:** SPR, sensor, biosensor, light source, optical device

## Abstract

The notion of surface plasmon resonance (SPR) sensor research emerged more than eight decades ago from the first observed phenomena in 1902 until the first introduced principles for gas sensing and biosensing in 1983. The sensing platform has been hand-in-hand with the plethora of sensing technology advancement including nanostructuring, optical technology, fluidic technology, and light source technology, which contribute to substantial progress in SPR sensor evolution. Nevertheless, the commercial products of SPR sensors in the market still require high-cost investment, component, and operation, leading to unaffordability for their implementation in a low-cost point of care (PoC) or laboratories. In this article, we present a comprehensive review of SPR sensor development including the state of the art from a perspective of light source technology trends. Based on our review, the trend of SPR sensor configurations, as well as its methodology and optical designs are strongly influenced by the development of light source technology as a critical component. These simultaneously offer new underlying principles of SPR sensor towards miniaturization, portability, and disposability features. The low-cost solid-state light source technology, such as laser diode, light-emitting diode (LED), organic light emitting diode (OLED) and smartphone display have been reported as proof of concept for the future of low-cost SPR sensor platforms. Finally, this review provides a comprehensive overview, particularly for SPR sensor designers, including emerging engineers or experts in this field.

## 1. Introduction

Surface plasmon resonance (SPR) has drawn enormous attention and found extensive applications in chemical sensors and biosensors. The notion of SPR sensor research began more than 80 years ago from the first observed phenomena by Wood [1], until the initially introduced principle for gas sensing and biosensing by Liedberg et al. [2]. This milestone is quite progressive among other sensing platform developments, particularly in its prominent features such as its sensitivity, real-time detection, and label-free assay. The fundamental principle of SPR sensor construction lays on the resonance of a strong electromagnetic field oscillation at the interface of nanometal film and a dielectric medium with p-polarized incident light resulting in a dark band profile in the light reflectivity at a specific wavelength and incident angle. To date, the past three decades have witnessed remarkable improvements and several implementations of the SPR sensor technology including in the area of chemical sensing, foodborne marker screening, environmental monitoring and medical diagnostics [3,4,5].

Light technology has been through a long evolution since more than a century ago marked by the invention of incandescent light and a halogen lamp [6]. Laser subsequently emanated and profoundly explored into a more complex integration such as in the development of helium-neon laser as the first gas laser at Bell Labs [7] and the semiconductor laser diode at General Electric using GaAs material [8]. Later on, the light emitting diode (LED) technology proposed by Oleg Vladimirovich Losev in 1927 was introduced as modern lighting and gained tremendous attention despite its drawbacks from monochromatic color and low emission flux [9], and the unfavorable impacts of silicon-based materials yielding only the red or green color spectra due to the limited band gap energy properties. However, revolutionary research on the building-up of blue LED based on III-V compound semiconductor by Nakamura successfully exploited the ability of LED devices to emit all primary colors (red, green and blue/RGB) [10,11]. Henceforth, the organic light-emitting diode (OLED) appeared as a promising technology with the ease of process and surpasses the other light technologies, such as laser, in regards to room temperature processes and the possibility for fabrication on large and flexible substrates [12,13,14], enabling the integration of red, green, and blue colors into a single device to obtain visible range spectrum [15].

In building an SPR sensing platform, the light source is one of the critical factors contributing to the ultimate sensing performance, configuration, optical design, and real-time data acquisition methodology. In fact, the advancement of light source technology for SPR sensor development, distinctively for the configurations, sensing principles, and miniaturization and portability designs have made this topic widespread and exceedingly enticing. In this article, a thorough summary of the physical basis of light sources for SPR sensors is focused through a broad and varied cross-section of the relevant literature. We endeavor to offer a comprehensive understanding from the history of light source technology to the latest progress and trends on light source-related SPR sensor research with a brief general overview on surface plasmon (SP) excitation configuration and measurement setup.

## 2. SPR Sensor Configuration

Fundamentally in SPR sensor construction, the resonance of SP is the key towards high performance. The resonance condition at the interface of a thin metal and dielectric medium can be coupled optimally by p-polarized light in a particular wavelength and an incident angle. It occurs when the wave vector value of the SP wave is identical to the propagation constant of the incident light. To get a profound understanding of this phenomenon, the SP wave vector (*β*_SP_) in nanometal film surface is postulated in the following equation [5]:

(1) βSP=Re{2πλεMεDεM+εD}

where *λ* is the wavelength of the incident light; while *ε*_M_ and *ε*_D_ are the real part value of metal and medium dielectric constant, respectively. Another essential factor is the propagation length (*L_X_*) of the SP wave expressed by the following formula:

(2) $$L_{X}=\frac{\pi }{2\lambda }\frac{\varepsilon_{iM}}{\varepsilon_{M}^{2}}{\left(\frac{\varepsilon_{M}\varepsilon_{D}}{\varepsilon_{M}+\varepsilon_{D}}\right)}^{\frac{3}{2}}$$

where the *ε*_*i*M_ is the imaginary part value of the metal-dielectric constant.

In the SP generation, a particular wavelength requires a specific configuration to match the light propagation constant of the incident light at a precise angle of incidence. The light source preference determines the priority of exploration and modification of the whole sensor configuration. For example, by using the polychromatic light source, the wavelength interrogation is necessary to obtain the SPR reflectivity. In contrast, in the monochromatic light source application, the angular interrogation for the SPR reflectivity profile plot is more critical.

### 2.1. Grating

**Figure 1:**
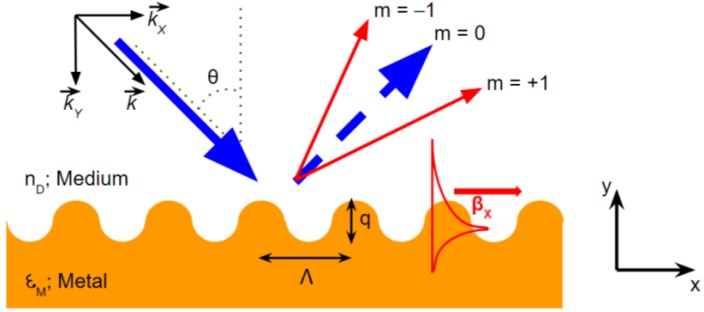
The configuration of the SP grating coupler.

Grating-enhanced SPR excitation has been a notable pioneer in SPR configuration. The grating configuration of SP excitation is depicted in Figure 1. Wood presented the first phenomena of SPR excitation on the grating configuration in 1902 [1]. Later on, improvements were carried out until Rayleigh developed an analytical solution to the Wood’s anomalies that describes the diffraction angles involved in grating techniques using the following formula [16]:

(3) $$\sin\;\left(\theta m\right)=\sin\;\theta +m\frac{\lambda }{\Lambda }$$

where θ is the incidence angle of p-polarized light, θm is the diffraction angle, λ is the wavelength of incident light, and Λ is the groove period. This description allows the calculation of diffraction angle of any scattered order from the grating period m, the incidence angle of light (θ) and the wavelength (λ).

The passing-off of the order n happens when sin (θ*m*) = ±1. Therefore, from Equation (3), the wavelengths of a spectrum generating the passing-off of a diffracted order are given by [17]:

(4) $$n\frac{\lambda }{\Lambda }=-\sin(\theta m)\pm 1;\;m=\pm 1,\;\pm 2,\;\pm 3,\;\ldots$$

The wavelength of the passing-off described above is called Rayleigh wavelength.

**Figure 2:**
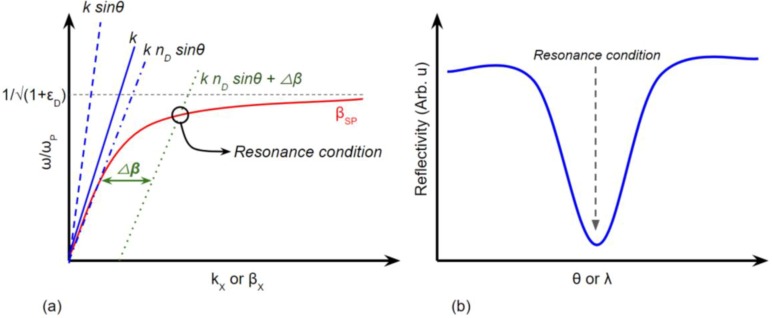
(**a**) Dispersion relation of grating coupler-based SP excitation. (**b**) Reflectivity profile of light due to SPR absorption.

The relation of incident light vector and grating configuration in resonance condition can be expressed by [18]:

(5) nD sinθ+mλΛ=±Re {εMεDεM+εD}+Δnef

where *n*_D_ is the refractive index (RI) of the medium and $\Delta n_{ef}=Re\;\left\{\Delta \beta \frac{\lambda }{2\pi }\right\}$, and Δβ is the propagation constant shifting in the presence of the grating structure. The dispersion relation curves of the grating coupler based-SPR excitation are presented in Figure 2.

### 2.2. Prism Coupler

Surface plasmon excitation using prism coupler was introduced by Kretschmann and Otto in 1968 [19,20].

**Figure 3:**
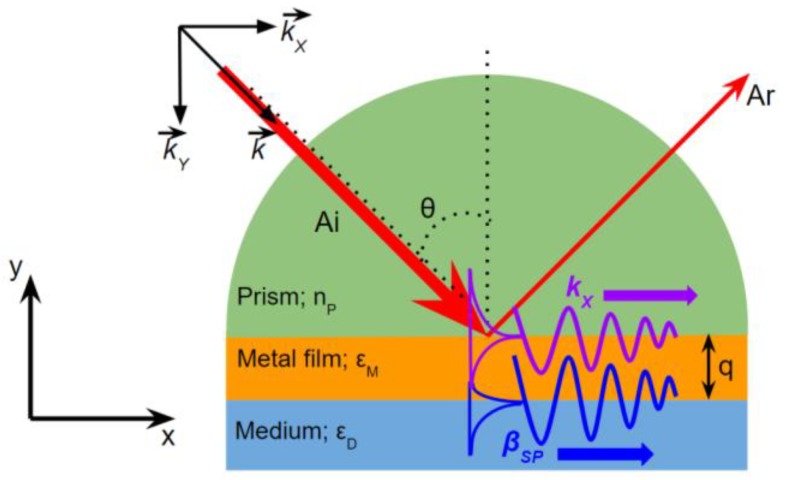
SPR excitation by prism coupling using Kretschmann configuration.

The prism coupling based on Kretschmann configuration [19,21] has become a standard technique to excite the SP in regards to its alignment simplicity leading to easily controlled parameter and variable (Figure 3). The underlying principle of this is to conduct the excitation of SP using transverse magnetic (TM) wave (*k*) to the smooth metal sensing nanofilm (*ε*_M_) through a high RI prism with its larger RI than the RI of the medium (*n*_P_ > *n*_D_) in a specific incident angle (θ).

The resonance condition of the prism coupler configuration is described by:

(6) $$k_{X}=\beta_{\mathrm{SP}}$$

(7) 2πλ⋅nP⋅sinθ=2πλ⋅Re {εMεDεM+εD}

Therefore, the incident angle of SPR can be calculated using the following equation:

(8) θ=sin −1 [(1nP)⋅Re {εMεDεM+εD}]

**Figure 4:**
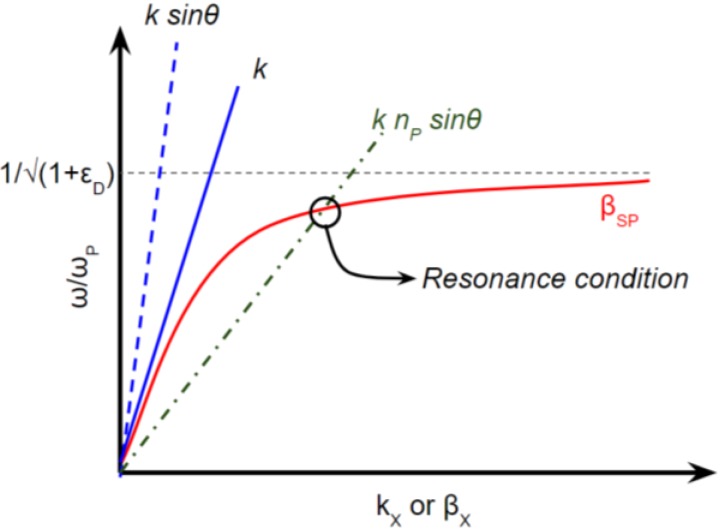
Dispersion relation of TM incident light coupling SP.

The dispersion relations of prism coupler-based SP excitation is illustrated in Figure 4. It is seen that by using a refractive index material *n*_P_, the propagation constant of incident light *k* is able to couple the wave vector of surface plasmon *β*_SP_ in the intersection point representing the resonance condition.

### 2.3. Waveguide

**Figure 5:**
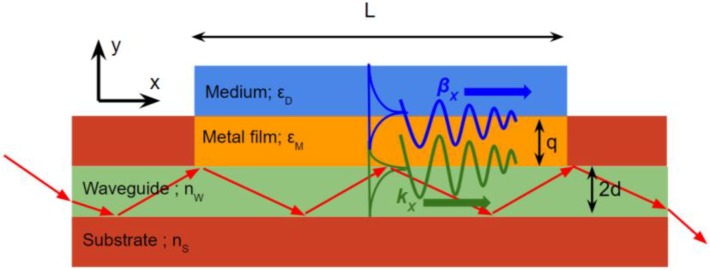
SPR coupling using waveguide structure. TIR of TM waves the waveguide layer excites SP in the metal film region L.

A guided mode in the SPR planar substrate to improve the SPR sensor performance is noticed as one of the convincing strategies. Waveguide coupler SPR is generally constructed in the homogenous high RI material $n_{W}$ in the limited thickness (2d), and sandwiched with the substrate or cladding layer to perform the total internal reflection (TIR) wave phenomena along the waveguide layer. To gain insight into the planar waveguide concept in SPR sensor configuration, a waveguide structure for SP excitation is described in Figure 5.

Note that although the SP can be excited when the propagation constant of the coupling wave in the x-direction, $k_{X}$ is equal to the wavevector of SP ($\beta_{X}$). Like in the principles of prism couple explained above, the planar waveguide configuration is unable to interrogate the incident angle scanning. As a consequence, the wavelength interrogation method is the only option for the signal acquisition technique. Alternatively, the waveguide thickness (2d) can be adjusted for the optimum numerical aperture to obtain the resonance condition considering that the numerical aperture of the waveguide thickness (2d) represents the incident angle coupling of the TM wave below the metal sensing.

**Figure 6:**
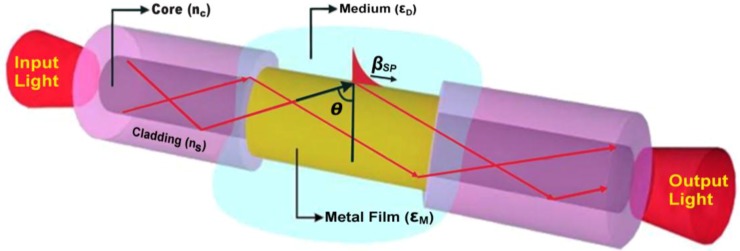
SP excitation using optical fiber structure.

Lavers and Wilkinson proposed the first waveguide-based SPR sensor in 1994 for the liquid sample medium [22]. Interestingly, the waveguide structure for SP excitation can also be constructed from the optical fiber in which a particular region of the fiber cladding is removed by etching. The primary sensing element in this combined fiber-planar configuration is the surface plasmon wave (SPW) with its hybrid nature, which consists of the guided mode coupled to a surface plasmon polariton. Next, in the core interface, the thin metal film is deposited surrounding the core. The waveguide structure consisting of the optical fiber is illustrated in Figure 6. Here, the oscillation of a surface plasmon wave (SPW) occurs along the fiber core or a waveguide layer of the planar structure. Its slightly different refractive index in comparison with the guided mode, enhanced by the structure without metal layer, makes it work effectively. In this configuration, the integration of numbers of sensing element on a single device is possibly realized for the fabrication of highly integrated, multichannel and robust sensing devices. Modifications of sensing element are hugely varied, such as by microfabrication in parallel or series where wavelength division multiplexing techniques extract signals from different sensing elements.

The SP coupling using optical fiber has offered several advantages over the conventional waveguide. First, the cylindrical waveguide of the optical fiber waveguide enables the TE or TM wave considering that polarization lights of any direction easily trigger the SP excitation. Second, one may notice that total dimension of the fiber optic waveguide-based sensor is small, yet, the active surface sensing area is more extensive because the tube-like surface of the metal as the sensing membrane accommodates more volume of sample as depicted in Figure 6. Nevertheless, a waveguide structure for SP excitation holds drawbacks in the sensitivity performance of random numerical aperture (NA) dependent-incident angle θ. Another significant issue is related to the non-adjustable incident angle which makes polychromatic light the only suitable light source to obtain SPR reflectivity profile. Consequently, the angular interrogation method is not applicable for the measurement methodology.

### 2.4. Localized SPR

There has been a tremendous amount of interests in noble metal behavior since the 4th century AD, proven by the invention of Lycurgus cup [23]. The term localized surface plasmon resonance (LSPR) is coined from the ability of these materials to convert energy, mainly from photons into a collective oscillation of conduction band electrons.

The confinement of the plasmonic field in the nanoparticle or nanostructure of the noble metal is the heart of LSPR. In this LSPR confinement, the light extinction profile entirely depends on the nanoparticle material, size, shape, aspect ratio, and the interspaces to the neighboring nanoparticles/nanostructures [24,25].

The extinction E(λ) of the LSPR as the accumulation from the light absorption and scattering is described by [24,26]:

(9) $$E\left(\lambda \right)=\frac{24\;\pi^{2}N\;a^{3}\varepsilon_{D}^{3/2}}{\lambda ln\left(10\right)}\left[\frac{\varepsilon_{i}}{{\left(\varepsilon_{r}+\xi \varepsilon_{D}\right)}^{2}+\varepsilon_{i}^{2}}\right]$$

where the *ε*_i_ and *ε*_r_ are the imaginary and real part values of the metal-dielectric constant, respectively. *ε*_D_ is the dielectric constant value of the medium surrounding the nanoparticles and *ξ* is the form factor that describes the aspect ratio of the nanoparticles. For spherical NP, *ξ* is equal to 2, and the higher the aspect ratio, the higher the value of *ξ* needed in the formula. N is the finite polarizable element, and a is the diameter size of the particle.

Despite its rapid progress and trends, the reproducibility of sensing fabrication remains challenging in LSPR development because of the aspect ratio and particle size dependence as seen in Equation (9) In regards to the latter factor, for metallic nanoparticles of a given shape and material composition, LSPR characters strongly rely on the particle size. Apart from the spheroidal nanoparticles, the non-spheroidal nanoparticles are difficult to measure, yet, experimental and electrodynamic simulations can still be performed and indicate the dependence of sensitivity on the particle shape.

The impact of particle shape is demonstrated by the work of Mock et al., which discovered that the spectra of silver nanoparticles of different shapes (spheres, triangles, and cubes) but similar volume were strongly linked with their structures [27]. In addition, regarding the shape of nanoparticles, sharp-tipped nanoparticles, such as nanopyramids, nanostars, nanotriangles, and other particular shapes, are prominently noticed to reinforce higher refractive index sensitivity due to the higher electromagnetic fields produced on their protruding parts and nanoparticles shape-based characterization is more accurate than that of an aspect ratio-based one. It is important to note that particles with sharp tips produce much higher refractive index sensitivities that would be predicted from their aspect ratios alone.

**Figure 7:**
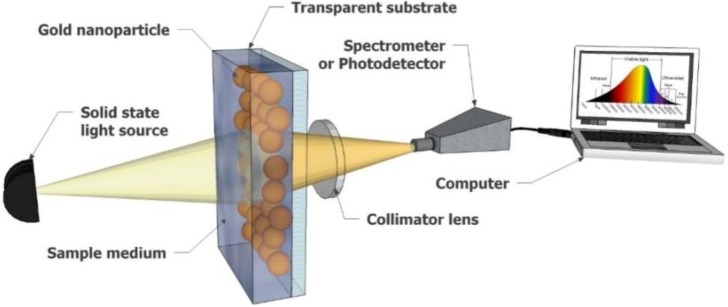
LSPR-based sensor using transmission attenuation configuration.

Another substantial element in the assembly of the LSPR sensor is the scattering factor in the attenuation extinction. Based on this parameter, the plasmonic excitation using polychromatic light is preferable than using the monochromatic type because of its more straightforward analysis. The configuration of the transmission-based LSPR sensor is depicted in Figure 7.

## 3. SPR Measurement, Methodology and Performance Parameters

The light source and wavelength preferences (polychromatic or monochromatic) are playing essential roles in SPR sensor development. The polychromatic light source can be described as the light source with broadband wavelengths along the spectrum. On the other hand, the monochromatic light source can be described as the light source that contains a single or very short band of wavelength. The example of a monochromatic light source is laser, whether He-Ne laser or laser diode. While, the example of polychromatic light sources are sunlight, incandescent light, a halogen lamp, LED, and OLED.

In addition, the poly- or mono-chromatic light source preferences will be strongly correlated to the SPR signal measurement methodologies and the configuration of SP excitation structures. The SPR detection technique will be translated in the sensorgram for real-time signal interpretation.

**Figure 8:**
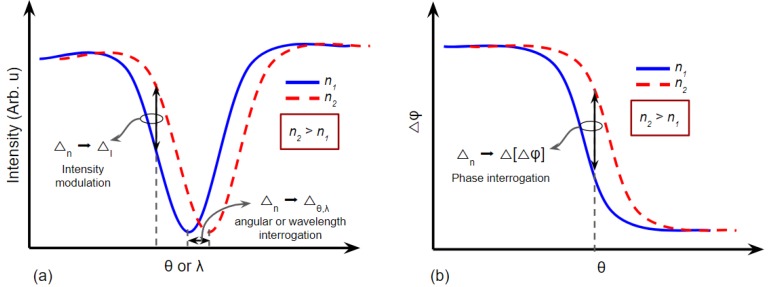
The methodology of SPR measurement (**a**) principle of intensity, angular, and wavelength interrogation. (**b**) The principle of phase interrogation method for SPR signal acquisition.

There are four types of methodology to measure the SPR signal. The first one is intensity modulation, wherein this method, the principle is to detect the intensity change of the reflectivity in particular incident angle or wavelength (Figure 8a). Pertaining to the array detector, this methodology is beneficial to obtain an image using an SPR sensor or commonly called as SPR imaging (SPRi). In SPRi, the observation of the contrast image in the sensing region can be presented in video or photograph. The second methodology is incident angle interrogation. In this method, monochromatic light is applied as the light source to couple SP; therefore, SPR condition from different RI medium is the contribution of various incident angles (θ).

The angular (or incident angle) and wavelength interrogation of SPR can be deviated based on the type of light source to couple the SPR. In the case of using the monochromatic light (single wavelength), such as laser, the angular interrogation method is required. While in the case of polychromatic light for fixation of incident angle, the wavelength interrogation method is necessary. The angular and wavelength interrogation methods for SPR measurements are illustrated in Figure 8a.

Another methodology for SPR signal acquisition is the phase interrogation method (Figure 8b). It is explicitly used for the coherent monochromatic light source in SPR instrumentation. Nevertheless, this method needs phase shift equipment, such as a lock-in amplifier. Moreover, the optical setup of this method is more complicated in comparison with the other three methodologies. This drawback results in a small number of studies reported this method for SPR sensor device, especially in the commercial product in the market.

**Figure 9:**
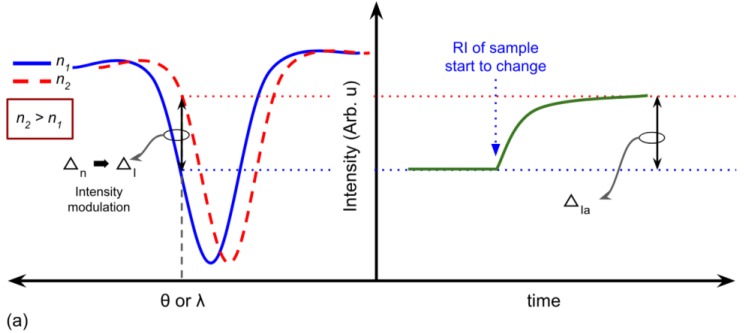

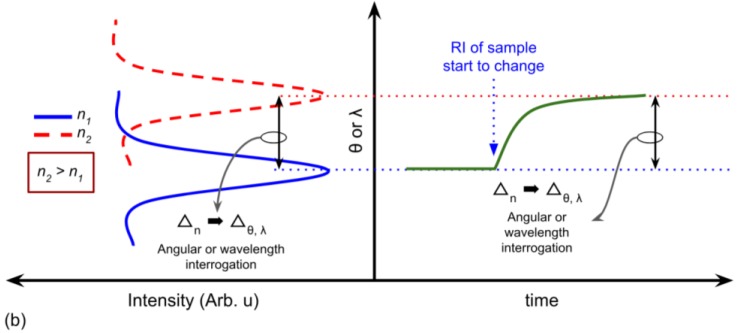
Sensogram curve plot from the SPR measurement methodology by (**a**) intensity modulation and (**b**) angular or wavelength interrogation.

One of the SPR sensor advantages is a real-time measurement of the refractive index shift in the medium near the sensing surface. In this measurement, the time axis is plotted on the sensorgram graph to monitor the SPR signal obtained from the methodology mentioned in Figure 8. The data processing of sensorgram is illustrated in Figure 9.

There are several parameters to indicate the performance of the SPR sensor during the measurement and detection. The first one is sensitivity, which is determined by the ratio of SPR signal magnitude (output signal) compared to the measurand (∆_RI_ or ∆_C_). This parameter is correlated with the slope of the calibration curve [22]. Sensitivity (S) in general is expressed as S_RI_ = ∂Y/∂*n* and S_C_ = ∂Y/∂*c*, where Y is the output signal, *n* and *c* are the measurand input indicating refractive index values and concentration, respectively. S_RI_ and Sc is the sensitivity in terms of different refractive indices and concentrations of the measurand, respectively. The next parameter is resolution (r_Sensor_) defined as the smallest change of sample resulting in the detectable output signal r_Sensor_ = *σ*/S_RI_, where $r_{\mathrm{Sensor}}$ is the resolution of SPR sensor, σ is the noise of the output signal, and $S_{\mathrm{RI}}$ is the sensitivity of the sensor performance in terms of refractive index values shifting [5,18].

Another critical parameter is the limit of detection (LOD). The general definition of LOD is the smallest concentration or quantity giving the output signal in the new measurement equal to the three times of standard deviation (or noise 3*σ*) [28]. It can be expressed as LOD = 3*σ*/S, where S is the sensitivity of the sensor in any terms, whether RI, concentration, or molar. Lastly, one of sensing parameter is the dynamic range (D). Dynamic range is determined by the measurand input, how large the ∆_RI_ or ∆_C_ to influence the signal output of the SPR sensor. In general concept of the sensor technology, including SPR sensor, there is a trade-off correlation between D and S. The higher the D value, the smaller the S value, and vice versa [29,30].

## 4. Light Source and Platform Preferences for SPR Sensor

**Figure 10:**
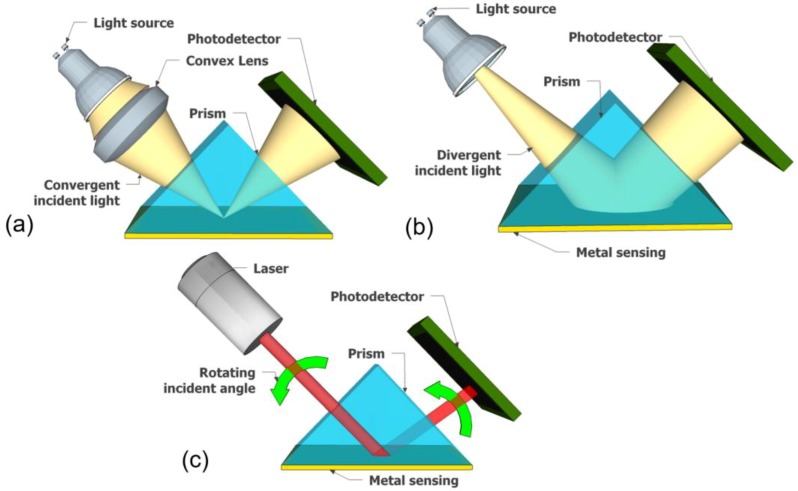
Optical configuration of incident light in SPR sensor apparatus in Kretschmann configuration. (**a**) Convergent, (**b**) divergent, (**c**) rotating for incident angle scanning.

The type and size of the light sources can be a significant consideration for a particular configuration of SPR sensor platform, whether using the conventional or miniaturized platform, fixed or portable apparatus, with its pros and cons of the features. In addition, the spectral color preference plays a key role in determining the detection method, whether using intensity modulation, wavelength or phase interrogation. It also defines the configuration of the incident light to excite surface plasmon in the sensing metal. Pertaining to the monochromatic light source (laser), a broader possibility for the incident light configuration is offered—whether convergent, divergent or rotating incident light (Figure 10)—where it is noticed that this configuration is closely related to the detection method preference, in particular for the incident angle interrogation. In contrast, for polychromatic light sources, the rotating incident light is not preferable, because the incident light will be dependent on *ε*_M_(λ) and *ε*_D_(λ). Consequently, as the polychromatic light contains several wavelength values, various incident angles will be correlated to different wavelengths in obtaining resonance condition. Therefore, the incident angle interrogation method is hard to be applied using the polychromatic light source. Furthermore, the complexity of the light sources alignment and the drawbacks are presented in Table 1 as a general background and comparison.

### 4.1. Incandescent Lamp

The incandescent lamp is the light source used in the first observation of surface plasmon phenomena in 1902 by Wood, and its explanation was described in details and published by Lord Rayleigh five years later [1,31]. It can be understood that in that era, the incandescent lamp was the only mature technology available for the light source experiment. In the later technology, the halogen lamp, categorized as the incandescent lamp with a tungsten filament, is still employed as the polychromatic wavelength source in the SPR sensing configuration. Halogen lamp technology is superior regarding the broad spectrum of the light wavelength. Therefore, in the fix incidence angle configuration and wavelength interrogation acquisition methods, this light source is preferable.

The halogen lamp has been used for SPR sensor platform based on uniform-waist tapered optical fibers and reflective elements. The sensitivity obtained through the wavelength interrogation technique of about 10^−4^ refractive index units per nanometer (RIU/nm) has been claimed [32]. Lin et al. proposed the side-polished multimode optical fiber and applied halogen light source for SPR sensor configuration. The sensing performance with the resolution of 3 × 10^−6^ RIU was achieved [33]. Another SPR configuration using optical fiber modified by cold plasma was proposed by the same group to amplify the biosensing performance [34]. The biomolecular sample of 5 ng bovine serum albumin (BSA) was successfully detected on the metal sensing.

The concept of grating-based SPR imaging (SPRi) using a halogen lamp was introduced in 2006 for the detection of BSA [35]. Hastings et al. proposed a halogen lamp guided by optical fiber to couple the SPR using the Kretschmann configuration to enhance the long and short range SPR mode due to the broadband wavelength of the incident light [36]. Kazuma et al. explored the use of halogen light for LSPR coupling by high refractive index prism. The resolution performance of the system and the limit of detection (LOD) achieved 2.8 × 10^−4^ RIU and 0.3 µg/mL, for bulk RI sample and streptavidin, respectively [37]. LSPR based biosensor utilizing commercial halogen lamp and spectrometer system for the detection of DNA hybridization has demonstrated an LOD of 1 nM [38]. Commercially available and low-cost halogen lamps as the excitation source are proposed for the LSPR excitation in AgAu nanorings. It was claimed that by using AgAu nanorings, the LSPR enhancement reached 4.3 and 4.7 fold higher compared to the utilization of Au and Ag nanospheres, respectively [39].

Large metal nanowire array was proposed by Ngoc et al. to elicit the LSPR phenomena using commercial white light tungsten. Besides, this structure is noticed to contribute to the enhancement factor in surface-enhanced Raman scattering (SERS). Interestingly, it was described that the coupled-mode LSPR wavelength with the nanogap of around 20 nm between adjacent metal nanowires could be precisely tuned to match the resonance condition at any excitation source in the range of 500 to 1000 nm [40]. Slavik et al. introduced a miniaturized light source implemented in long-range SPR (LRSPR) using a Teflon AF thin layer under the metal sensing. This sensing structure induced a double symmetrical surface plasmon wave. The dynamic range performance of 8 × 10^−3^ RIU was achieved [41]. The summary of SPR sensors using a halogen l amp as the light source is presented in Table 2.

### 4.2. Laser

After its invention in 1958, several elaborative research, analytical and applications of laser technology, monochromatic light, and the optical designs were reported. A decade later, Kretschmann and Otto, proposed two similar techniques of SP excitation in the silver film through the high refractive index prism by monochromatic light from laser light source [20,42]. Subsequently, some scientists published the proposed methods either for fundamental experimental works or theories related to the SP excitation using a prism coupler [43,44]. Using laser as a light source in SPR configuration, the incident angle scanning method of SPR reflectivity is applied in regards to its monochromatic light characteristics. Based on the technology of the lasing mechanism, this section is written to mainly elucidate two type of lasers: gas laser and a laser diode. The gas laser mechanism fundamentally makes use of electric current in gas medium to be discharged and to produce a coherent light [8]. Whereas, for the laser diode, the electric current accumulation in the quantum well results in stimulated emission of photons. While the gas laser is recognized as the conventional technology and known for its bulky size and high power operation laser, the laser diode contrarily offers benefits such as the possibility for miniaturization and low power consumption as the impact of the applied semiconductor technology.

#### 4.2.1. Gas Laser

The common type of gas sensor in the market is known to be the helium-neon laser (He-Ne laser) [7,45]. This laser medium consists of 85% helium and 15% neon inside the gain medium. The first SPR sensor principle for gas detection was proposed using a He-Ne laser in 1982 [46]. Liedberg et al. demonstrated the SPR sensor for the detection of halothane gas and the IgG protein interaction on the surface of the gold metal film [2,46] as the first SPR biosensor using a laser.

The dark field SPR spectroscopy utilizing a He-Ne laser was introduced in microarray sensing membrane. This method has potential for biosensor applications, where the affinity reactions can be real-time monitored simultaneously for high-throughput sample measurement [47]. A metal sensing design based on the theory of distributed Bragg reflector (DBR) for vertical cavity surface emission laser (VCSEL) was proposed for better full-width half-maximum (FWHM) and dynamic range of the SPR sensor. A 632 nm-wavelength laser on a Kretschmann configuration was applied to demonstrate the proof of concept and resulting in a resolution value of 1.28 × 10^−5^ RIU [48]. Yusmawati et al. conducted an analysis of commercial carbonated drink using He-Ne laser based SPR sensor. The detection limit of sugar contents from 0.01 to 0.05% was achieved successfully [49].

Furthermore, an LSPR imaging configuration illuminated by He-Ne laser was reported without any prisms for the coupling technique. The detection sensitivity up to 16.6% of transmitted intensity per RIU was demonstrated [50]. A Kretschmann configuration was modified for the waveguide optical sensor coupled by the green light from a He-Ne laser. The sensing film structure from porous anodic alumina/aluminum (PAA/Al) multilayer film was utilized for the Au monolayer replacement. Finally, the reflectivity profile improvement was proven in the presented report [51]. Turker et al. reported the grating-based SPR sensor using a He-Ne laser and photodiode. The transmission based plasmonic mechanism was utilized as the sensing mechanism. The sensing performance with a long-term refractive index noise down to 6.3 × 10^−6^ RIU/√Hz was achieved [52]. In addition, a nanohole rectangular film was utilized for the transmitted LSPR sensor by the He-Ne laser excitation. While a 2D CCD camera was used for the photodetector. The sensing resolution down to 6.4 × 10^−6^ RIU was reached for the bulk RI measurement. Lertvachirapaiboon et al. reported research on grating-based LSPR sensor using a He-Ne laser (632.8 nm). A gold grating film was fabricated by the imprinting process that is resulting in an angle of incidence at 47°. This study focuses on the simplicity of the imprinted sensing fabrication although the full-width half-maximum (FWHM) of the reflectivity profile is broader as being compared to the evaporated sensing film [53]. An argon-ion laser emitting light at 1550 nm wavelength was applied in a tilted fiber Bragg grating (TFBG) as an SPR sensor apparatus for the detection of intact epithelial cells as analytes in cell suspensions. The achieved detection limit was lower than 2.0 × 10^6^ cells/mL in the proposed apparatus [54]. Another study reported the utilization of He-Ne laser as a light source in SPR sensor apparatus for NH_3_ gas detection. The sensing films of tin oxide (SnO_2_) as the NH_3_ gas sensitive membrane in the Kretschmann configuration were characterized [55]. Next, another Kretschmann-based SPR sensor configuration applying He-Ne laser light source was proposed for the detection of *N. meningitidis* DNA. The sensing film was a 200 nm of ZnO on a gold film, and the sensing performance was shown by the sensitivity of 0.03°/(ng/μL) and LOD of 5 ng/μL [56].

Kim et al. reported FO-SPR sensor for a label-free immunosensor for blood detection of patients afflicted with Alzheimer’s disease. In this study, a He-Ne laser with 632.8 nm of wavelength was implemented for the light excitation source. The target sample was fibrinogen in blood plasma. This study reported a detection limit of 20 ng/mL fibrinogen concentration in patient’s plasma blood [57]. Heating effects from the different power of He-Ne laser in a Kretschmann-based SPR sensor were observed and analyzed. The red (1.5 mW) and green (15 mW) lasers were compared and resulted in a local increment of temperature at the laser spot position below ∼0.1 K and ∼1 K for red and green laser, respectively. It was concluded that the modification of silica glass refractive index results in the order of 10^−5^–10^−6^ RIU; and this range of shifting value could be considered negligible concerning the excitation of SPR sensor [58].

#### 4.2.2. Laser Diode

Laser diode for the SPR sensor was proposed firstly in 1988 by Matsubara et al. [59]. The configuration harnessed a lens to obtain a convergent incident light with angularly spread beam to the metal sensing through a Kretschmann prism. A photodiode array was used to capture the divergent beam of the reflected light; consequently, the reflectivity profile could be analyzed using Fourier transform optics.

Spectroscopic imaging based-SPR sensor was presented for binding events monitoring receptor array on disposable sensor chips. This developed system employed a laser diode and showed superior performance in comparison with the LED light source [60]. Zhang et al. proposed a simple, stable, and high-resolution SPR sensor using a laser diode and quadrant cell photodetector. The light beam was focused through a high refractive index prism on a thin Au film divided into two areas, detection and reference area [61]. The dual wavelengths intensity modulation method for Kretschmann based SPR sensor was introduced using laser diodes. The LOD of 2 × 10^−6^ RIU was obtained by the proposed technique [62]. A GeAs laser diode with a wavelength of approximately 670 nm was constructed in a commercial SPR sensor (NanoSPR, Chicago, IL, USA). The IgG protein detection on the functionalized polymer sensing layer was demonstrated with a sensing regeneration capability [63]. A quantum cascade laser (QCL) in the mid-infrared range was prepared for the SPR sensor for CO_2_ detection. Referring to a He-Ne laser light source, the sensor performance improvement in the visible range was recorded [64]. Patskovsky et al. proposed the use of Si prism in Kretschmann structure with a laser diode for the SPR sensor light source. A detection limit of about 10^−6^ RIU was demonstrated in the detection of Ar and N_2_ gas [65]. A laser diode in a portable SPR sensor with a rotating mirror was conducted in the study of protein interaction. The palm-size platform is potential for the practical use of field experiments. The detection limit of 2.5 × 10^−6^ RIU was resulted [66]. Fast detection SPR imaging based on the laser beam illumination was configured using the 637 nm diverging light from a laser diode. The intensity modulation interrogation method was applied in order to obtain an image. The brightness image profile of the sensing area represents the absorption of the analyte in the metal sensing interface. The LOD of 5 × 10^−6^ RIU was the outcome of the proposed configuration [67]. Dual wavelength laser diode (658 and 980 nm) were generated for the self-referencing SPR-biosensors. By using the accumulation of the differential signals between two reference wavelengths, the influence of the bulk refractive index can be suppressed up to 20 times, and the final signal predominantly reflects the surface processes [68]. A laser diode (976 nm) was noted to be integrated into an SPR sensor based on fiber Bragg Grating (FBG). The light from a diode laser was guided by an optical fiber such as a photonic crystal device. By the wavelength division multiplexing (WDM), the 976 nm of the light source was shifted to 1060 nm. The Yb^3+^ was measured as the active medium. Moreover, it is notable that the FBG plays an important role like a cavity in a photonic crystal fiber [69]. The summary of laser-based SPR sensors is listed in Table 3.

### 4.3. Polychromatic Solid State Lighting

The polychromatic solid-state lighting technology in this review is categorized into three subsections; the first is a light-emitting diode (LED), the second is the organic light-emitting diode (OLED). From the technology perspective, the laser diode is included in solid-state lighting. However, due to its monochromatic wavelength output, it is reviewed in the laser technology part. In the third subsection, the SPR sensor configuration in smartphone platforms is reviewed. The smartphone-based SPRs typically applies a light source excitation from the smartphone component, categorized as polychromatic solid-state lightings, such as display screen and flashlight.

The main advantage of the polychromatic solid-state lighting technology in SPR sensor is its small size, low-cost, and low power consumption which make it a potential contributor in the development progress of portable platform of SPR sensor with a wavelength interrogation method.

#### 4.3.1. Light Emitting Diode

Light-emitting diode offers a broader wavelength spectrum of the output light in comparison with laser. Therefore, a wavelength interrogation method for the SPR signal acquisition can be used for the measurement technique. In addition, the required wavelength to obtain the resonance condition, particularly for the gold monolayer sensor, is in the area of red in the case of the visible wavelength range. Hence, for the SPR apparatus development, LED is hitherto one of the favorite light sources besides halogen lamp and laser. The advantages are owing to the fact where firstly, the higher the technology maturity, the greater the availability in the market. The LED device with a red color wavelength can be constructed with a silicon-based LED [70]. Notably, in the early 90 s when SPR sensors were early developed and commercialized, the red color LED technology was mature enough and considered as a low-cost light source [71]. Secondly, using solid-state lighting, such as LED, the SPR sensing apparatus can be efficiently designed towards miniaturization and portability for broader applications. After the invention of the blue and white color LED, various designs of SPR configurations were reported [72,73].

Melendez et al. demonstrated an SPR sensor apparatus for the commercial purpose of utilizing a near-infrared LED light source. The group discovered a potential use of LED as a light source in SPR apparatus which surpassed the properties of the impractical and bulky laser. The low cost LED light path was focused by the lens and coupling the sensing metal with a Kretschmann prism. The SPR sensor resolution of 10^−5^ RIU was achieved [74]. Later, this LED-based SPR configuration had turned into a commercial product, Spreeta™ from Texas instrument (Dallas, TX, USA) [75].

An LED light source with a wavelength of 820 nm was mounted for the SPR light source, while the laser diode was also used in the system to produce light scattering signals. However, the concentration suspension of bacterial spores of 10^7^ mL^−1^ was detected insignificantly [73]. Ho et al. proposed the white LED light sources for the halogen lamp replacement in an SPR sensor apparatus. By harnessing the broad spectral light source, the wavelength interrogation method is highly suitable for the data acquisition. The measurement error of 1.98 × 10^−4^ RIU was demonstrated for the measured sample of 147 ppm glycerin in water [76]. An enticing structure was constructed by Akimoto et al. where a portable SPR sensor using a small probe was coupled with an LED. The sensor probe was constructed with a 5-cm glass cylinder, 1.5 mm in the diameter. A beam splitter and polarizer were then employed to guide and polarize the light to the photodiode. The resolution value of 1.2 × 10^−5^ RIU RI and the detection limit of 50 ng/mL denoted the sensor performance for bulk refractive index sample [77]. Suzuki et al. signified the synergistic impacts of an SPR sensor with waveguide coupling and dual LED as the light sources. The signal acquisition method summed up two differential intensities at two wavelengths to improve the SNR. This remarkable miniaturized device was carried out for the measurement of BSA immunoreaction. The proposed device performed outstandingly with the LOD of 2.3 × 10^−5^ RIU [78]. Another dual color LEDs were applied in the miniaturized SPR sensor. This proposed architecture made a potential use of nanohole array grating and transmission spectral measurement. The intensity modulation in the specific wavelengths was recorded as the resonance signals. The LOD of 6 × 10^−4^ RIU was reported [79].

In another instance, unique microcontact-printed protein patterns were proposed for SPRi with a LED light source. The patterning was completed by polydimethylsiloxane (PDMS) stamping. A dynamic range of IgG protein detection of 0.005 to 0.5 mg/mL were successfully resulted [80]. An exciting SPR sensor apparatus was brought up by Ng et al. using warm white light LED and phase interrogation for signal acquisition. The LOD below 10^−7^ RIU was reached for the measurement of the NaCl solution [81]. Multispectral SPRi was also built up using five color LEDs integrated into an optical fiber and illuminating the sensing layer through a Kretschmann configuration. By performing a DNA hybridization measurement, an impressive LOD of 3 × 10^−6^ RIU had been achieved [82]. Another captivating notion implemented a commercial LED light source in an SPR sensor for the Hg^2+^ detection in pure and tap water, respectively. A highly unique sequence of ssDNA probes were immobilized on the gold surface to capture the Hg ions in the T-rich ssDNA specifically. The proof of concept with the detection limit of 0.01 ng/mL was shown [83].

Huang et al. did one of the ideas combining a microfluidic device with an LSPR sensor. In this arrangement, Gold nanoparticle arrays significantly induced a scattering phenomenon where the spectra could be shifted due to the change of refractive index in the medium. The sensing performance was noticed by the 10^−4^ RIU resolution of glycerol and an LOD of approximately 270 ng/mL of anti-biotin [84]. A similar method of exploiting LSPR scattering phenomena was introduced by Aslan et al. using the LED light source. The colloids of gold nanoparticles were confined in the poly-lysine coated well, and the collimator collected the transmitted light to the fluorimeter. The IgG immunoreaction was run on the platform, and a detection limit of 0.05 µg/mL was obtained [85]. Another noteworthy approached was demonstrated by Mitsushio et al. where a glass fiber based SPR sensor was integrated with LED as the light source. The fundamental concept was the position of a photodiode detector at the end of the waveguide to analyze the transmitted intensity modulation of the light. The detection limit of 1 × 10^–4^ RIU was reported [86]. Other progress is shown in the detection study of RBL-2H3 cells in a miniaturized, low-cost, and portable SPR sensor with a waveguide coupling. A detection limit of 1.65 × 10^−3^ RIU was successfully recorded [87]. Myriads of techniques to achieve lower LOD were developed, such as shown by a comprehensive work of Chuang et al., who elaborated a disposable and low-cost SPR sensor with a microfluidic cartridge for the detection interferon gamma (IFN-γ), a biomarker for tuberculosis. In the measurement range of IFN-γ from 0.01 to 100 nM/mL, the detection limit of 10 pM was achieved [88]. In addition, Cetin et al. imparted the idea of constructing an LSPR sensor with nanohole gratings in a handheld-sized device. The application of a LED light source in the configuration of transmittance measurements and the CMOS photodetector under the plasmonic gratings played the critical role in this work. A resolution sensor of 4 × 10^−3^ RIU is greatly reported [89]. The summary of SPR sensor review utilizing LED is presented in Table 4.

#### 4.3.2. Organic Light-Emitting Diode

Organic electronics development has been attracting great attention from scientists for modern electronics applications including the development of organic transistors, display, lighting technology, and sensors [90]. Organic light-emitting diodes (OLEDs) as the latest generation of light source device were also employed in several optical sensors technology including the SPR sensor platforms. OLED has been advantageous to be integrated into the bio-optical sensor. First, it is due to the low-cost fabrication process which makes use of the organic materials and room temperature process [90]. Second, in regards to the biodegradability of the organic material, hence, the use of this device for bio-application component significantly supports the significant and essential features and enables disposable usage [90]. The third, from the optical platform perspective, in OLED technology, self-healing effect is not an issue like in the LED technology. This feature is the consequence of the large-sized OLED substrate (glass or flexible plastic) which is useful for the heat dissipation layer [14,91]. Subsequently, from the structure point of view, OLED leads to the simple alignment of light source excitation, particularly in SPR sensor platform [92].

The first OLED-based SPR sensor platform was developed by Frischeisen et al. for the measurement of NaCl solution. This group presented OLED with three different colors to cover the whole visible light spectrum and fascinatingly discovered that OLED offers a tremendous potency for miniaturization of SPR sensor platform. The LOD of this structured device was 6 × 10^−4^ RIU [92]. Prabowo et al. continued the development of Frischeisen group’s OLED platform by harnessing a brightness enhancement film and reflective polarizer in the incident light instead of in the reflection light [93]. The LOD improvement of 2.6 × 10^−4^ RIU has been achieved using bimetallic sensing structure and white OLED [94]. This group utilizes this OLED-based SPR sensor platform for various biomedical applications, such as for DNA detection from clinical samples [95], DNA hybridization [96], viral particle quantification [97,98], and protein marker detection [97]. The list of OLED-based SPR sensor is listed in Table 5.

#### 4.3.3. Smartphone-Based SPR Sensor

An advanced integrated SPR sensor configuration with a smartphone as the main component has emanated as an appealing approach towards smart-sensor technology. The display or flashlight built-in camera can be utilized as the SPR light source. Preechaburana et al. proposed a small and portable SPR sensor utilizing an iPhone device. The light source excitation was taken from the iPhone screen programmed into a red color, and the Kretschmann prism was fabricated and customized using PDMS material. The reflection light path was guided to the front camera of the iPhone. The signal acquisition method was set using the intensity modulation of the red color. The resolution performance of 2.14 × 10^−6^ RIU was reportedly achieved [99]. Another research conducted by Liu et al. presented an optical fiber-based SPR using a smartphone. The flashlight was applied for the light source excitation, while the primary camera near the flashlight was utilized as the photodetector. Three channels of optical fiber were configured as a measurement channel, reference channel, and control channel. The resolution performance of 7.4 × 10^−5^ RIU was acquired and resulting in the detection limit of 47.4 nM of IgG measurement [100]. Another noteworthy work demonstrated the integration of LSPR based sensor with a smartphone camera as a photodetector. A customized additional optical module was fabricated, and a commercial broad spectral light was utilized as an excitation source to illuminate the gold nanoparticle colloidal solution in a cuvette. A detection limit of 19.2 µg/mL and 25.7 µg/mL were attained for the BSA and Trypsin measurement, respectively [101]. Wang et al. demonstrated the transmission grating-based SPR sensor in a smartphone platform. The changing of the refractive index sample was indicated by the shifting color in the smartphone camera as a photodetector. In the proposed platform, an extended optical module consisting of LED light and the transmission grating were utilized. The handheld sensor system was employed in urine sample measurement. This platform achieves an LOD of 0.01 mg/mL of BSA target detection referring to up to 30 times improvement in comparison with the commercial portable sensor [102]. The summary of SPR sensor configuration utilizing smartphone as the main platform is listed in Table 6.

## 5. The Summary, Future Perspectives, and Challenges

The trend of SPR sensor development based on light source technology has been reviewed. The SPR sensor has been implemented in various fields, from biomedical, food sciences, environmental monitoring, toxic or chemical compound detection, pharmacy, and industry. The light source and photonic technology has been developing for decades and influencing the SPR sensor configuration, design, and features, for particular required applications, for example, the construction of a large-size design for sensitive experimental detection mostly conducted at conventional laboratory or the building up of a portable size design which is suitable for experimental target where the sensitivity performance is not primarily critical parameter. When it comes to an ultralow detection limit as the spotlighted performance of SPR sensor, medical application is one of the most demanding fields requiring the feature, such as in early detection of biomarkers. Meanwhile, for industrial applications, another performance parameter such as wide dynamic range will be more significant, for instance, in oil contamination and other environmental monitoring. Besides, mono- or poly-chromatic light source features are also a prominent component to determine the detection methodology using an SPR sensor. Based on the review, the light source is not the only critical component in SPR sensor configuration and performance enhancement. The selection of the detector component, detection method, the design of sensing film structure, the surface chemistry technology, and the fluidic chip are also crucial to enhance either sensitivity, detection limit or dynamic range performance of the SPR sensor. Nevertheless, since the SPR sensor is an optical sensor based on photonic excitation, all the exquisite components of the sensor will not be optimum without the great stability of the applied light source. The employment of the proper light source technology is the first critical factor in the establishment of SPR sensor design.

Nanoelectronics technology and manufacture are rapidly growing towards advanced technology to target smaller device, such as in the display panel, photonic devices, memory devices and digital circuits. This technology has been noted as a substantial trajectory towards future sensing technology which makes miniaturized and integrated SPR sensor device well-positioned to be regarded as the critical manufacturing technologies and trends in the future. Throughout the journey, smartphone-based SPR sensor development has marvelously marked a significant milestone of the digital technology integration to biosensor application technology and lab on chip platform [101,102]. The brilliant concept reflected in this integration accounts the future trend of SPR sensing technology which is small, portable, handheld, smart, user-friendly, easy to store, offers easy data transmittance, and potentially involves artificial intelligence software to simplify the signal acquisition, as well as data analysis. Disposability and biodegradability of a sample container encompass a challenging yet beneficial feature for fluidic chip technology in an SPR sensor platform. Besides, a very slow flow-rate and a smaller sample volume for real-time detection are of good prospects for more profound development.

Furthermore, these advancements may entail image processing technology, which impacts future trends of SPR imaging applications either in real time (video) or non-real time (image). This technology does not merely improve on the binding status but also upgrades the morphology, binding distribution, and high throughput analysis. To support high-quality imaging, the wide area, stable, and reliable light source technology is indispensable. We envisage the future development of organic light source as an alternative solution for this feature necessity [103] beside another feature such as disposability and simple alignment for modularity. The availability of small and high-resolution camera technology in the market, such as a built-in camera in a smartphone, implies a powerful trigger for progress. For instance, with the existing camera with CCD (charge-coupled devices) or CMOS (complementary metal-oxide-semiconductor) technology fabricated in a small device. These technologies remarkably paved a path towards advantageous feature for a highly collimated light for analysis in miniaturized SPR sensor platform.

To an infinite extent, internet-of-things (IoT) technology will be actively involved in the data communication of the SPR sensor in clinical settings. The measurement of the clinical sample by the paramedics can be completed altogether with diagnostics procedures based on the transmitted measurement data. Then, a remote analysis can be performed by an expert, such as in a hospital office or directly in a medical doctor’s device. This application is greatly beneficial for the laboratory with high biosafety level to reduce the risk of contamination.

The main challenges of the research and development in SPR sensors are the high-cost platforms and components. Usually, the commercial platforms do not meet the affordability requirements of small research groups or points of care (PoC) to invest and do the maintenance. Therefore, it is a potential roadmap in the future for scientists and engineers in academic fields and industry to develop a low-cost yet highly performed SPR sensor platform.

For application outlooks, the detection of a marker target in undiluted samples, such as in milk [104], blood [105,106], urine, or saliva is highly prospective as well as in the screening of pollutants and hazards pertaining to sustainability and environmental conservation study. For this purpose, an elaborative surface chemistry technology is a great match, such as by the application of polymer-based surface chemistry or zwitterionic technology to perform a new trend of antifouling binding in the complex medium [104,107,108,109].

## Figures and Tables

**Table 1:** Light source features and their implementation in SPR sensor platforms.

No	Feature	Incandescent Lamp	Gas Laser	Laser Diode	LED	OLED
1	Size	Bulky	Bulky	Small	Small	Small
2	Color	Polychromatic	Monochromatic	Monochromatic	Polychromatic	Polychromatic
3	Disposability	No	No	Yes	Yes	Yes
4	Detection method possibility	λ int., intensity mod.	θ int., intensity mod., phase int.	θ int., intensity mod., phase int.	λ int., intensity mod.	λ int., intensity mod.
5	Incident light	Convergent, divergent	Convergent, rotating	Convergent, rotating	Convergent, divergent	Convergent, divergent
6	Component of optical alignment	Lens, fiber optic	Lens, fiber optic Motor	Lens, fiber optic Motor	Lens, fiber optic	Microstructure film
7	Light source alignment	Hard	Hard	Moderate	Moderate	Simple
8	Drawback	Self-heating, size, lifetime, stability	Self-heating, size, stability	Self-heating, stability	Self-heating, stability	Technology maturity

**Table 2:** The summary of SPR sensor development utilizing halogen lamp technology.

No	Configuration	Technical Remarks	Target Sample	Results/Performance	Ref.
1	Optical fiber	Uniform-waist tapered optical fibers	Water	Sensitivity: 10^−4^ RIU	[32]
2	Optical fiber	Side-polished multimode optical fiber	Water, ethanol, DNA	Sensitivity: 3 × 10^−6^ RIU	[33]
3	Optical fiber	Cold plasma modified Side-polished optical fiber	BSA	5 ng	[34]
4	Grating	Grating based SPRi	BSA	∼225 spots cm^−2^	[35]
5	Kretschmann	Optical fiber coupled-prism to enhance long and short range SPR	Streptavidin	LOD: 2.3 × 10^−5^, RIU: 11 pg/mm^2^	[36]
6	LSPR	Ag nanospheres and nanorods on TiO_2_ substrate	Streptavidin	LOD: 2.8 × 10^−4^, RIU: 0.3 µg/mL	[37]
7	LSPR	Au nanosphere for the detection of DNA mutation in Roundup Ready soybean	DNA hybridization	LOD: 1 nM DNA	[38]
8	LSPR	LSPR excitation in AgAu nanorings	Methylene blue.	4.3 and 4.7 fold LSPR enhancement	[39]
9	LSPR	Large metal nanowire array	Air	SERS enhancement	[40]
10	LRSPR	Kretschmann, long-range SPR sensing structure (Teflon AF under Au layer).	Water-diethylene glycol	Dynamic range: 8 × 10^−3^ RIU	[41]

**Table 3:** The summary of SPR sensor development with laser technology.

No	Laser Type	Technical Remark	Target Sample	Performance	Ref.
1	He-Ne laser	Kretschmann, SPRi, λ = 632.8 nm, microarray sensing membrane	Si coating, SAM	Decay length ~4 µm	[47]
2	He-Ne laser	Kretschmann, dielectric mirror TiO_2_/SiO_2_ sensing structure, λ = 632	Glucose solution	Res: 1.28 × 10^−5^ RIU Dynamic range: 1.331–1.50 RIU	[48]
3	He-Ne laser	Kretschmann, angular int., λ = 632.8 nm	Sugar content in carbonated drink	LOD: 0.01–0.05%	[49]
4	He-Ne laser	LSPRi, λ = 632.8 nm, nanohole arrays.	SAM	detection sensitivity ~16,6%/RIU	[50]
5	He-Ne laser	Waveguide, green light (λ = 534.5 nm), PAA/Al sensing structure.	Fe(II) solution	n/a	[51]
6	He-Ne Laser	Grating, transmission measurement, λ = 632.8 nm, integrated flow cell and detector	NaCl solution	Res: 6.3 × 10^−6^ RIU/√Hz	[52]
7	He-Ne laser	Imprinted AuNP Grating, λ = 632.8 nm.	Fe(II)-BTP and PEDOT:PSS	n/a (proof of concept)	[53]
8	Argon-ion laser	Tilted fiber Bragg grating (TFBG), λ = 1550 nm	Epithelial cells	LOD ~2 × 10^6^ cells/mL	[54]
9	He-Ne laser	Kretschmann, Au/SnO_2_ sensing film, angular int., λ = 633 nm	Ammonia gas	Sensitivity 0.055°/ppm (0.5–250 ppm)	[55]
10	He-Ne laser	Kretschmann, Au/ZnO sensing film, angular int., λ = 633 nm	DNA of *N. meningitidis*	LOD: 5 ng/μL	[56]
11	He-Ne laser	Optical fiber coupling, Intensity modulation, λ = 632.8 nm	Fibrinogen on plasma blood	LOD: 20 ng/mL	[57]
12	He-Ne laser and Laser diode	Kretschmann, angular int., He-Ne laser: 1.5 mW, red, λ = 632.8 nm; Laser diode: 15 mW, green, λ = 543 nm	Air, thermal effects on laser spot area.	Local thermal drift: red laser ∼0.1 K and green laser ∼1 K	[58]
13	Laser diode	Kretschmann with rotating diffuser, SPRi, CCD camera detector, λ = 633 nm, high throughput and disposable sensing design.	IgG, BSA	Proof of concept for multi-sample detection.	[60]
14	Laser diode	Kretschmann, linear laser incident, two channels detection area, quadrant cell photodetector.	Pb^2+^ ions	~0.2 nM or 0.04 ppb	[61]
15	Laser diode	Kretschmann, intensity mod. at dual wavelengths references.	DNA hybridization	LOD: 2 × 10^−6^ RIU	[62]
16	Laser diode	Integratted in NanoSPR™	IgG	Sensing regeneration	[63]
17	Quantum cascade laser	Kretschmann CaF_2_ prism, TiO_2_/Au layer on sensing, angular int., λ = 633 nm.	CO_2_	5 times sensitivity improvement	[64]
18	Laser diode	Kretschmann, Si prism, λ = 1200 nm for air medium, λ = 1500 nm for aqueous medium, phase modulation.	Ar and N_2_	LOD: 10^−6^ RIU	[65]
19	Laser diode	Portable, Kretschmann, rotating mirror for the incident angle adjustment, powered by battery, 2 channels measurement.	PSA	LOD: 2.5 × 10^−6^ RIU	[66]
20	Laser diode	Kretschmann, angular int., diverging laser beam, λ = 637 nm	Ethanol solution	LOD: 5 × 10^−6^ RIU	[67]
21	Laser diode	Dual wavelengths, λ = 658 nm and 980 nm, self-referencing	Diluted NaCl on PBS	20 times reducing noise from bulk RI medium effect	[68]
22	Laser diode	FBG based coupling, λ = 976 nm	Yb^3+^	n/a	[69]

**Table 4:** The summary of the LED light source in SPR sensor platforms.

No	LED Type	Technical Remark	Target Sample	Performance	Ref.
1	NIR LED	Additional laser diode for scattering enhancement, λ = 820 nm.	Bacterial spores	LOD: 10^7^ mL^−1^	[73]
2	White LED	Kretschmann, wavelength int., 200 µm pin hole to control incident light.	Glycerin solution	Res: 1.98 × 10^−4^ RIU	[76]
3	LED	Waveguide coupling using 5 cm cylindrical glass probe (diameter 1.5 mm), photodiode detector, wavelength int.	Glycerin, BSA	Res: 1.2 × 10^−5^ RIU LOD: 50 ng/mL	[77]
4	Dual LED	Waveguide coupling using 5 cm flat pyrex glass, intensity mod. At two wavelengths, optional LED for different wavelength.	Ethanol solution, BSA	LOD: 2.3 × 10^−5^ RIU	[78]
5	Dual LEDs	Transmission grating based, nanohole array sensing, intensity mod.	Biotin-streptavidin binding	LOD: 6 × 10^−4^ RIU	[79]
6	LED	Kretschmann, SPRi, intensity mod., patterned sensing, λ = 648 nm	Cholera toxin (CT), IgG	IgG detection range 0.005 to 0.5 mg/mL	[80]
7	Warm white light LED	Dual Kretschmann (for reference and target sample), phase int. in preferred wavelength.	NaCl solution	Res: 10^−7^ RIU	[81]
8	Five color LEDs	Kretschmann, SPRi, optical fiber waveguide, wavelength int.	DNA hybridization	Res: 3 × 10^−6^ RIU	[82]
9	White LED	Kretschmann, wavelength int., integrated with confocal microscope	Mercury ion	LOD: 0.01 ng/mL	[83]
10	LED	LSPR, integrated with microfluidic device, transmittance measurement, intensity mod.	Glycerol, biotin-antibiotin	LOD: 10^−4^ RIU LOD: 270 ng/mL	[84]
11	LED	LSPR, transmittance measurement, intensity mod.	IgG	LOD: 0.05 µg/mL	[85]
12	LED	Glass fiber waveguide, intensity mod.	Benzyl alcohol in methanol	LOD: 10^−4^ RIU	[86]
13	LED	Miniaturize, low-cost, and portable platform, waveguide coupling, wavelength int.	RBL-2H3 cells	LOD: 1.65 × 10^−3^ RIU	[87]
14	LED	Portable, integrated with microfluidic chip, low-cost, disposable, aptamer probe.	Interferon-γ	LOD: 10 pM	[88]
15	LED	Transmission grating, nanohole array	IgG, BSA	Res: 4 × 10^−3^ RIU	[89]

**Table 5:** The summary of OLED light source in SPR sensor platforms.

No	Light Source	Technical Remark	Target Sample	Performance (LOD)	Ref.
1	Three colors OLED	Kretschmann, light source attachment on prism, wavelength int.	NaCl solution	6 × 10^−4^ RIU	[92]
2	Red and green OLED	Kretschmann, OLED attachment on prism, red and green OLED, brightness and reflective polarizer enhancement, intensity modulation At dual wavelengths.	Sucrose water, IgG	3 × 10^−6^ RIU 40.6 pg/mL	[93]
3	White color OLED	Kretschmann, OLED attachment on prism, wavelength int., brightness and reflective polarizer enhancement, bimetallic film, intensity modulation at dual wavelengths.	Sucrose water, IgG	2.6 × 10^−6^ RIU 40.3 pg/mL	[94]
4	Tunable color OLED	Kretschmann, integration of intensity modulations.	EV71 viral particle, VP1 protein	67 vp/mL; 4.8 pg/mL	[97]
5	Red color OLED	Kretschmann, intensity modulation at dual wavelengths.	IS6110 DNA	63 pg/mL	[95]
6	Red color OLED	Four layer structures, Kretschmann, intensity modulations at dual wavelengths.	EV71 viral particle	43 vp/mL	[98]
7	Red color OLED	Graphene layer, π-π stacking ssDNA-graphene, AuNP signal enhancement.	DNA hybridization	28 fM	[96]

**Table 6:** The summary of smartphone-based SPR sensor platforms.

No	Platform	Technical Remark	Target Sample	Performance	Ref.
1	iPhone	PDMS Kretschmann prism, light source from iPhone screen, the front camera as a photodetector, optional sensing plate using commercial CM5.	β2 microglo-bulin	Res:2.14 × 10^−6^ RIU LOD: 0.1 mg/mL In serum; 0.25 mg/mL in urine	[99]
2	Smartphone	Optical fiber coupling, three channels, main camera as photodetector	IgG	Res:7.4 × 10^−6^ RIU, LOD: 47.4 nM	[100]
3	Optical module and iPhone integration	White light source, LSPR, transmission illumination, extended optical module, main camera as photodetector, AuNP colloid solution in cuvette.	BSA, Trypsin	LOD: 19.2 µg/mL (BSA); 25.7 µg/mL (Trypsin)	[101]
4	Optical module and iPhone integration	LED light source, transmission grating, extended optical module, main camera as a photodetector.	Urine, BSA	0.01 mg/mL	[102]

## References

[1] Wood R.W. (1902). On a remarkable case of uneven distribution of light in a diffraction grating spectrum. Philos. Mag. Ser. 6.

[2] Liedberg B., Nylander C., Lunström I. (1983). Surface plasmon resonance for gas detection and biosensing. Sens. Actuators B.

[3] Šípová H., Homola J. (2013). Surface plasmon resonance sensing of nucleic acids: A review. Anal. Chim. Acta.

[4] Piliarik M., Párová L., Homola J. (2009). High-throughput SPR sensor for food safety. Biosens. Bioelectron..

[5] Homola J. (2008). Surface plasmon resonance sensors for detection of chemical and biological species. Chem. Rev..

[6] Furfari F.A. (2001). A Different Kind of Chemistry: A History of Tungsten Halogen Lamps. IEEE Ind. Appl. Mag..

[7] Javan A., Bennett W.R., Herriott D.R. (1961). Population inversion and continuous optical maser oscillation in a gas discharge containing a He-Ne mixture. Phys. Rev. Lett..

[8] Hecht J. (2010). Short history of laser development. Opt. Eng..

[9] Zheludev N. (2007). The life and times of the LED—A 100-year history. Nat. Photonics.

[10] Nakamura S., Mukai T., Senoh M. (1994). Candela-class high-brightness InGaN/AlGaN double-heterostructure blue-light-emitting diodes. Appl. Phys. Lett..

[11] Pimputkar S., Speck J.S., DenBaars S.P., Nakamura S. (2009). Prospects for LED lighting. Nat. Photonics.

[12] Lee S.M., Kwon J.H., Kwon S., Choi K.C. (2017). A Review of Flexible OLEDs Toward Highly Durable Unusual Displays. IEEE Trans. Electron Devices.

[13] Tang C.W., Vanslyke S.A. (1987). Organic electroluminescent diodes. Appl. Phys. Lett..

[14] Maindron T., Templier F. (2014). OLED: Theory and Principles. OLED Microdisplays: Technology and Applications.

[15] Fung M.-K., Li Y.-Q., Liao L.-S. (2016). Tandem Organic Light-Emitting Diodes. Adv. Mater..

[16] Kooyman R.P.H., Schasfoort R.B.M., Tudos A.J., Schasfoort R.B.M., Tudos A.J. (2008). Physics of Surface Plasmon Resonance. Handbook of Surface Plasmon Resonance.

[17] Maystre D. (2012). Theory of wood’s anomalies. Springer Ser. Opt. Sci..

[18] Homola J., Homola J. (2006). Surface Plasmon Resonance Based Sensors.

[19] Kretschmann E., Raether H. (1968). Radiative decay of non-radiative surface plasmons excited by light. Z. Naturforsch..

[20] Otto A. (1968). Excitation of nonradiative surface plasma waves in silver by the method of frustrated total reflection. Z. Phys..

[21] Kretschmann E. (1972). Decay of non radiative surface plasmons into light on rough silver films. Comparison of experimental and theoretical results. Opt. Commun..

[22] Lavers C.R., Wilkinson J.S. (1994). A waveguide-coupled surface-plasmon sensor for an aqueous environment. Sens. Actuators B Chem..

[23] Gartia M.R., Hsiao A., Pokhriyal A., Seo S., Kulsharova G., Cunningham B.T., Bond T.C., Liu G.L. (2013). Colorimetric Plasmon Resonance Imaging Using Nano Lycurgus Cup Arrays. Adv. Opt. Mater..

[24] Willets K.A., Van Duyne R.P. (2007). Localized surface plasmon resonance spectroscopy and sensing. Annu. Rev. Phys. Chem..

[25] Stewart M.E., Anderton C.R., Thompson L.B., Maria J., Gray S.K., Rogers J.A., Nuzzo R.G. (2008). Nanostructured plasmonic sensors. Chem. Rev..

[26] Anker J.N., Hall W.P., Lyandres O., Shah N.C., Zhao J., Van Duyne R.P. (2008). Biosensing with plasmonic nanosensors. Nat. Mater..

[27] Mock J.J., Hill R.T., Tsai Y.J., Chilkoti A., Smith D.R. (2012). Probing dynamically tunable localized surface plasmon resonances of film-coupled nanoparticles by evanescent wave excitation. Nano Lett..

[28] Long G.L., Winefordner J.D. (1983). Limit of Detection A Closer Look at the IUPAC Definition. Anal. Chem..

[29] Li Z., Ligthart L.P., Huang P., Lu W., Van Der Zwan W.F. Trade-off between sensitivity and dynamic range in designing digital radar receivers. Proceedings of the 2008 International Conference on Microwave and Millimeter Wave Technology.

[30] Chen P., Shu X., Cao H., Sugden K. (2017). High-sensitivity and large-dynamic-range refractive index sensors employing weak composite Fabry-Perot cavities. Opt. Lett..

[31] Strutt J.W. (1907). On the Dynamical Theory of Gratings. Proc. R. Soc. Lond. A Math. Phys. Eng. Sci..

[32] Esteban O., Díaz-Herrera N., Navarrete M.-C., González-Cano A. (2006). Surface plasmon resonance sensors based on uniform-waist tapered fibers in a reflective configuration. Appl. Opt..

[33] Lin H.-Y., Tsao Y.-C., Tsai W.-H., Yang Y.-W., Yan T.-R., Sheu B.-C. (2007). Development and application of side-polished fiber immunosensor based on surface plasmon resonance for the detection of *Legionella pneumophila* with halogens light and 850 nm-LED. Sens. Actuators A Phys..

[34] Lin Y.-C., Tsao Y., Tsai W.-H., Hung T.-S., Chen K.-S., Liao S.-C. (2008). The enhancement method of optical fiber biosensor based on surface plasmon resonance with cold plasma modification. Sens. Actuators B Chem..

[35] Singh B., Hillier A.C. (2006). Surface plasmon resonance imaging of biomolecular interactions on a grating-based sensor array. Anal. Chem..

[36] Hastings J.T., Guo J., Keathley P.D., Kumaresh P.B., Wei Y., Law S., Bachas L.G. (2007). Optimal self-referenced sensing using long- and short- range surface plasmons. Opt. Express.

[37] Kazuma E., Tatsuma T. (2013). Localized surface plasmon resonance sensors based on wavelength-tunable spectral dips. Nanoscale.

[38] Jang H., Kwak C.H., Kim G., Kim S.M., Huh Y.S., Jeon T.J. (2016). Identification of genetically modified DNA found in Roundup Ready soybean using gold nanoparticles. Microchim. Acta.

[39] Rodrigues T.S., da Silva A.G.M., de Moura A.B.L., Freitas I.G., Camargo P.H.C. (2016). Rational design of plasmonic catalysts: matching the surface plasmon resonance with lamp emission spectra for improved performance in AgAu nanorings. RSC Adv..

[40] Ngoc L.L.T., Jin M., Wiedemair J., Van Den Berg A., Carlen E.T. (2013). Large Area Metal Nanowire Arrays with Tunable Sub-20 nm Nanogaps. ACS Nano.

[41] Slavík R., Homola J. (2006). Optical multilayers for LED-based surface plasmon. Appl. Opt..

[42] Kretschmann E., Reather H. (1968). Radiative decay of nonradiative surface plasmon excited by light. Z. Naturf..

[43] Simon H.J., Mitchell D.E., Watson J.G. (1975). Surface plasmons in silver films—a novel undergraduate experiment. Am. J. Phys..

[44] Siegman A., Fauchet P. (1986). Stimulated Wood’s Anomalies on Laser-Illuminated. IEEE J. Quantum Electron..

[45] Antes L.L., Goldsmith J., McMahan W. (1964). Pulsed Helium-Neon Gas Laser Applications. IEEE Trans. Mil. Electron..

[46] Nylander C., Liedberg B., Lind T. (1982). Gas detection by means of surface plasmon resonance. Sens. Actuators.

[47] Grigorenko A.N., Beloglazov A.A., Nikitin P.I. (2000). Dark-field surface plasmon resonance microscopy. Opt. Commun..

[48] Lin C.-W., Chen K.-P., Hsiao C.-N., Lin S., Lee C.-K. (2006). Design and fabrication of an alternating dielectric multi-layer device for surface plasmon resonance sensor. Sens. Actuators B.

[49] Yusmawati W.Y., Chuah H.P., Mahmood M.Y.W. (2007). Optical Properties and Sugar Content Determination of Commercial Carbonated Drinks using Surface Plasmon Resonance. Am. J. Appl. Sci..

[50] Lesuffleur A., Im H., Lindquist N.C., Lim K.S., Oh S. (2008). Laser-illuminated nanohole arrays for multiplex plasmonic microarray sensing. Opt. Express.

[51] Yamaguchi A., Hotta K., Teramae N. (2009). Optical Waveguide sensor based on a porous anodic alumina/aluminum multilayer film. Anal. Chem..

[52] Turker B., Guner H., Ayas S., Ekiz O.O., Acar H., Guler M.O., Dâna A. (2011). Grating coupler integrated photodiodes for plasmon resonance based sensing. Lab Chip.

[53] Lertvachirapaiboon C., Yamazaki R., Pienpinijtham P., Baba A., Ekgasit S., Thammacharoen C., Shinbo K., Kato K., Kaneko F. (2012). Solution-based fabrication of gold grating film for use as a surface plasmon resonance sensor chip. Sens. Actuators B Chem..

[54] Malachovska V., Ribaut C., Wattiez R., Caucheteur C. (2015). Fiber-Optic SPR Immunosensors Tailored to Target Epithelial Cells through Membrane Receptors. Anal. Chem..

[55] Paliwal A., Sharma A., Tomar M., Gupta V. (2016). Surface plasmon resonance study on the optical sensing properties of tin oxide (SnO_2_) films to NH_3_ gas. J. Appl. Phys..

[56] Kaur G., Paliwal A., Tomar M., Gupta V. (2016). Detection of Neisseria meningitidis using surface plasmon resonance based DNA biosensor. Biosens. Bioelectron..

[57] Kim J., Kim S., Nguyen T.T., Lee R., Li T., Yun C., Ham Y. (2016). Label-Free Quantitative Immunoassay of Fibrinogen in Alzheimer Disease Patient Plasma Using Fiber Optical Surface Plasmon Resonance. J. Electron. Mater..

[58] Galvez F., De Lara D.P., Spottorno J., García M.A., Vicent J.L. (2017). Heating effects of low power surface plasmon resonance sensors. Sens. Actuators B Chem..

[59] Matsubara K., Kawata S., Minami S. (1988). A Compact Surface Plasmon Resonance Sensor for Measurement of Water in Process. Appl. Spectrosc..

[60] O’Brien I.I.M.J., Perez-Luna V.H., Brueck S.R.J., Lopez G.P. (2001). A surface plasmon resonance array biosensor based on spectroscopic imaging. Biosens. Bioelectron..

[61] Zhang H.Q., Boussaad S., Tao N.J. (2003). High-performance differential surface plasmon resonance sensor using quadrant cell photodetector High-performance differential surface plasmon resonance sensor using quadrant cell photodetector. Rev. Sci. Instrum..

[62] Zybin A., Grunwald C., Mirsky V.M., Wolfbeis O.S., Niemax K. (2005). Double-Wavelength Technique for Surface Plasmon Resonance Measurements: Basic Concept and Applications for Single Sensors and Two-Dimensional Sensor Arrays. Anal. Chem..

[63] Chegel V., Whitcombe M.J., Turner N.W., Piletsky S.A. (2009). Deposition of functionalized polymer layers in surface plasmon resonance immunosensors by in-situ polymerization in the evanescent wave field. Biosens. Bioelectron..

[64] Herminjard S., Sirigu L., Herzig H.P., Crottini A., Pellaux J., Gresch T. (2009). Surface Plasmon Resonance sensor showing enhanced sensitivity for CO_2_ detection in the mid-infrared range. Opt. Express.

[65] Patskovsky S., Song I., Meunier M., Kabashin A.V., Montréal É.P. (2009). De Silicon based total internal reflection bio and chemical sensing with spectral phase detection. Opt. Express.

[66] Shin Y., Min H., Jung Y., Hyun B. (2010). A new palm-sized surface plasmon resonance (SPR) biosensor based on modulation of a light source by a rotating mirror. Sens. Actuators B Chem..

[67] Karabchevsky A., Karabchevsky S., Abdulhalim I. (2011). Fast surface plasmon resonance imaging sensor using Radon transform. Sens. Actuators B Chem..

[68] Nizamov S., Mirsky V.M. (2011). Self-referencing SPR-biosensors based on penetration difference of evanescent waves. Biosens. Bioelectron..

[69] Hao C.J., Lu Y., Wang M.T., Wu B.Q., Duan L.C., Yao J.Q. (2013). Surface Plasmon Resonance Refractive Index Sensor Based on Active Photonic Crystal Fiber. IEEE Photonics J..

[70] Daldosso N., Pavesi L. (2009). Nanosilicon photonics. Laser Photonics Rev..

[71] Dupuis R.D., Krames M.R., Member S. (2008). History, Development, and Applications of High-Brightness Visible Light-Emitting Diodes. J. Lightwave Technol..

[72] Meléndez J., Carr R., Bartholomew D., Taneja H., Yee S., Jung C., Furlong C. (1997). Development of a surface plasmon resonance sensor for commercial applications. Sens. Actuators B Chem..

[73] Perkins E.A., Squirell D.J. (2000). Development of instrumentation to allow the detection of microorganisms using light scattering in combination with surface plasmon resonance. Biosens. Bioelectron..

[74] Melendez J., Carr R., Bartholomew D.U., Kukanskis K., Elkind J., Yee S., Furlong C., Woodbury R. (1996). A commercial solution for surface plasmon sensing. Sens. Actuators B Chem..

[75] Melendez J.L., Carr R.A., Keller R.C. (1999). Integrally Formed Surface Plasmon Resonance Sensor. U.S. Patent.

[76] Ho H.P., Wu S.Y., Yang M., Cheung A.C. (2001). Application of white light-emitting diode to surface plasmon resonance sensors. Sens. Actuators B.

[77] Akimoto T., Wada S., Karube I. (2008). A surface plasmon resonance probe without optical fibers as a portable sensing device. Anal. Chim. Acta.

[78] Suzuki A., Kondoh J., Matsui Y., Shiokawa S., Suzuki K. (2005). Development of novel optical waveguide surface plasmon resonance (SPR) sensor with dual light emitting diodes. Sens. Actuators B.

[79] Escobedo C., Vincent S., Choudhury A.I.K., Campbell J., Brolo A.G., Sinton D., Gordon R. (2011). Integrated nanohole array surface plasmon resonance sensing device using a dual-wavelength source. J. Micromech. Microeng..

[80] Wilkop T., Wang Z., Cheng Q. (2004). Analysis of micro-contact printed protein patterns by SPR imaging with a LED light source. Langmuir.

[81] Ng S.P., Wu C.M.L., Wu S.Y., Ho H.P. (2011). White-light spectral interferometry for surface plasmon resonance sensing applications. Opt. Express.

[82] Sereda A., Moreau J., Boulade M., Olivéro A., Canva M., Maillart E. (2015). Compact 5-LEDs illumination system for multi-spectral surface plasmon resonance sensing. Sens. Actuators B Chem..

[83] Zhang H., Yang L., Zhou B., Liu W., Ge J., Wu J., Wang Y., Wang P. (2013). Ultrasensitive and selective gold film-based detection of mercury (II) in tap water using a laser scanning confocal imaging-surface plasmon resonance system in real time. Biosens. Bioelectron..

[84] Huang C., Bonroy K., Reekmans G., Laureyn W., Verhaegen K., De Vlaminck I., Lagae L., Borghs G. (2009). Localized surface plasmon resonance biosensor integrated with microfluidic chip. Biomed. Microdevices.

[85] Aslan K., Geddes C.D. (2009). Wavelength-Ratiometric Plasmon Light Scattering-Based Immunoassays. Plasmonics.

[86] Mitsushio M., Higo M. (2011). A gold-deposited surface plasmon resonance-based optical fiber sensor system using various light-emitting diodes. Anal. Sci..

[87] Yanase Y., Araki A., Suzuki H., Tsutsui T., Kimura T., Okamoto K., Nakatani T., Hiragun T., Hide M. (2010). Development of an optical fiber SPR sensor for living cell activation. Biosens. Bioelectron..

[88] Chuang T.-L., Chang C.-C., Chu-Su Y., Wei S.-C., Zhao X., Hsueh P.-R., Lin C.-W. (2014). Disposable surface plasmon resonance aptasensor with membrane-based sample handling design for quantitative interferon-gamma detection. Lab Chip.

[89] Cetin A.E., Coskun A.F., Galarreta B.C., Huang M., Herman D., Ozcan A., Altug H. (2014). Handheld high-throughput plasmonic biosensor using computational on-chip imaging. Light Sci. Appl..

[90] Shinar R., Shinar J. (2009). Organic Electronics in Sensors and Biotechnology.

[91] Chung S., Lee J.-H., Jeong J., Kim J.-J., Hong Y. (2009). Substrate thermal conductivity effect on heat dissipation and lifetime improvement of organic light-emitting diodes. Appl. Phys. Lett..

[92] Frischeisen J., Mayr C., Reinke N.A., Nowy S., Brütting W. (2008). Surface plasmon resonance sensor utilizing an integrated organic light emitting diode. Opt. Express.

[93] Prabowo B.A., Chang Y.-F., Lee Y.-Y., Su L.-C., Yu C.-J., Lin Y.-H., Chou C., Chiu N.-F., Lai H.-C., Liu K.C. (2014). Application of an OLED integrated with BEF and giant birefringent optical (GBO) film in a SPR biosensor. Sens. Actuators B Chem..

[94] Prabowo B.A., Su L.-C., Chang Y., Lai H., Chiu N.-F., Liu K.-C. (2016). Performance of white organic light-emitting diode for portable optical biosensor. Sens. Actuators B.

[95] Prabowo B.A., Chang Y.-F.F., Lai H.-C.C., Alom A., Pal P., Lee Y.-Y.Y., Chiu N.-F.F., Hatanaka K., Su L.-C.C., Liu K.-C.C. (2018). Rapid screening of Mycobacterium tuberculosis complex (MTBC) in clinical samples by a modular portable biosensor. Sens. Actuators B Chem..

[96] Prabowo B.A., Alom A., Secario M.K., Masim F.C.P., Lai H.C., Hatanaka K., Liu K.C. (2016). Graphene-based Portable SPR Sensor for the Detection of Mycobacterium tuberculosis DNA Strain. Procedia Eng..

[97] Prabowo B.A., Wang R.Y.L., Secario M.K., Ou P.-T., Alom A., Liu J.-J., Liu K.-C. (2017). Rapid detection and quantification of Enterovirus 71 by a portable surface plasmon resonance biosensor. Biosens. Bioelectron..

[98] Prabowo B.A., Alom A., Pal P., Secario M.K., Wang R.Y.L., Liu K.C. (2017). Novel Four Layer Metal Sensing in Portable SPR Sensor Platform for Viral Particles Quantification. Proceedings.

[99] Preechaburana P., Gonzalez M.C., Suska A., Filippini D. (2012). Surface plasmon resonance chemical sensing on cell phones. Angew. Chem. Int. Ed. Engl..

[100] Liu Y., Liu Q., Chen S., Cheng F., Wang H., Peng W. (2015). Surface Plasmon Resonance Biosensor Based on Smart Phone Platforms. Sci. Rep..

[101] Dutta S., Saikia K., Nath P. (2016). Smartphone based LSPR sensing platform for bio-conjugation detection and quantification. RSC Adv..

[102] Wang X., Chang T.W., Lin G., Gartia M.R., Liu G.L. (2017). Self-Referenced Smartphone-Based Nanoplasmonic Imaging Platform for Colorimetric Biochemical Sensing. Anal. Chem..

[103] Reineke S., Lindner F., Schwartz G., Seidler N., Walzer K., Lüssem B., Leo K. (2009). White organic light-emitting diodes with fluorescent tube efficiency. Nature.

[104] Vaisocherová H., Ševců V., Adam P., Špačková B., Hegnerová K., de los Santos Pereira A., Rodriguez-Emmenegger C., Riedel T., Houska M., Brynda E. (2014). Functionalized ultra-low fouling carboxy- and hydroxy-functional surface platforms: functionalization capacity, biorecognition capability and resistance to fouling from undiluted biological media. Biosens. Bioelectron..

[105] Mani V., Chikkaveeraiah B.V., Patel V., Gutkind J.S., Rusling J.F. (2009). Ultrasensitive immunosensor for cancer biomarker proteins using gold nanoparticle film electrodes and multienzyme-particle amplification. ACS Nano.

[106] Chen H., Zhang M., Yang J., Zhao C., Hu R., Chen Q., Chang Y., Zheng J. (2014). Synthesis and characterization of antifouling poly(*N*-acryloylaminoethoxyethanol) with ultralow protein adsorption and cell attachment. Langmuir.

[107] Gobi K.V., Iwasaka H., Miura N. (2007). Self-assembled PEG monolayer based SPR immunosensor for label-free detection of insulin. Biosens. Bioelectron..

[108] Sin M.-C., Chen S.-H., Chang Y. (2014). Hemocompatibility of zwitterionic interfaces and membranes. Polym. J..

[109] Chen S., Li L., Zhao C., Zheng J. (2010). Surface hydration: Principles and applications toward low-fouling/nonfouling biomaterials. Polymer.

